# Assessment of the accuracy of malaria microscopy in private health facilities in Entebbe Municipality, Uganda: a cross-sectional study

**DOI:** 10.1186/s12936-021-03787-y

**Published:** 2021-06-06

**Authors:** Tobius Mutabazi, Emmanuel Arinaitwe, Alex Ndyabakira, Emmanuel Sendaula, Alex Kakeeto, Paul Okimat, Philip Orishaba, Simon Peter Katongole, Arthur Mpimbaza, Pauline Byakika-Kibwika, Charles Karamagi, Joan Nakayaga Kalyango, Moses R. Kamya, Grant Dorsey, Joaniter I. Nankabirwa

**Affiliations:** 1 School of Medicine, Makerere University College of Health Sciences, P.O. Box 7072, Kampala, Uganda; 2 Directorate of Medical Services, Special Forces Command, Uganda People’s Defence Forces, P.O. Box 11, Entebbe, Uganda; 3 Infectious Diseases Research Collaboration (IDRC), P.O. Box 7475, Kampala, Uganda; 4 Institute of Public Health and Management, Clarke International University, P.O. Box 7782, Kampala, Uganda; 5 Faculty of Health Sciences, Uganda Martyrs University, P.O. Box 5498, Kampala, Uganda; 6 Department of Medicine, University of California, San Francisco, San Francisco, USA

**Keywords:** Malaria, Diagnosis, Accuracy, Microscopy, Private health facilities

## Abstract

**Background:**

Although microscopy remains the gold standard for malaria diagnosis, little is known about its accuracy in the private health facilities in Uganda. This study evaluated the accuracy of malaria microscopy, and factors associated with inaccurate smear results at private health facilities in Entebbe Municipality, Uganda.

**Methods:**

Between April and May 2018, all patients referred for a malaria smear in 16 private health facilities in Entebbe municipality were screened, and 321 patients were enrolled. A questionnaire was administered to collect demographic and clinical information, facility-based smear results were recorded from the participant’s consultation notes, and a research slide was obtained for expert microscopy during exit interview. A health facility assessment was conducted, and information on experience in performing malaria microscopy was collected from all facility personnel reading smears and the data was linked to the participant’s clinic visit.

**Results:**

The test positivity rate of malaria parasitaemia was 15.0% by expert microscopy. The sensitivity, specificity and negative predictive value of the facility-based microscopy were high (95.8%, 90.1 and 99.2%, respectively). However; the positive predictive value (PPV) was low with 27/73 (63%) patients diagnosed with malaria not having the disease. Majority of the inaccurate results were from 2 of the 23 laboratory personnel reading the smears. The factors associated with inaccurate smear readings included being read by a technician; (1) who had less than 5 years’ experience in reading malaria smears (adjusted Odds Ratio [aOR] = 9.74, 95% confidence interval [CI] (1.06–89.5), p-value = 0.04), and (2) who was examining less than 5 smears a day (aOR = 38.8, 95% CI 9.65–156, p-value < 0.001).

**Conclusions:**

The accuracy of malaria microscopy in this setting was high, although one third of the patients diagnosed with malaria did not have the disease. Majority of the errors in smear readings were made by two laboratory personnel, with the main factor associated with inaccurate smear results being low experience in malaria microscopy. In-service training may be sufficient to eliminate inaccurate smear results in this setting, and these private facilities would be ideal model facilities to improve the quality of malaria microscopy in Uganda especially in the public sector where accuracy is still poor.

## Background

Despite the use of highly effective control interventions, malaria remains a significant health problem in many sub-Saharan countries [1]. In Uganda, malaria accounts for 27–34% of all outpatient visits, and 19–30% of all in-patient admissions [2]. Like many other endemic countries, Uganda has adopted the World Health Organization (WHO) recommendation for parasitological confirmation of malaria prior to treatment [3]. Data from the 2019 Uganda District Health Management Information System 2 (DHIS-2) showed that 71% of the reported malaria cases in the country had a laboratory confirmation [2]. Microscopy is currently the gold standard for malaria diagnosis in Uganda [3]. Using microscopy for laboratory confirmation of malaria has some advantages over rapid diagnostic tests including characterizing species, quantifying parasite density, assessing response to anti-malarial therapy and being cheaper if trained staff and equipment are available [4]. However, the accuracy of microscopy is limited by a number of factors including the parasite density, skill of the microscopist, age/immunity of the patient, and malaria endemicity [5–7]. The accuracy of the diagnostic used in the identification of malaria cases is crucial for case management, as it may affect the clinician’s treatment practices [3]. Studies have shown that although many of the clinicians use diagnostic results to guide malaria treatment, a number of times anti-malarials are prescribed in patients with negative test results [8–10]. One of the cited reasons for not adhering to test results to guide treatment practices is the fear of missing malaria cases due to false negative results [11–13]. This observation highlights the need for highly accurate diagnostics especially in areas where malaria transmission is declining so that the benefits of the malaria test and treatment recommendations can be realized.

Previous evaluations have shown that the accuracy of malaria microscopy is often poor [6], and some of the documented factors associated with inaccurate microscopy include; poorly organized health systems, poor supplies and lack of quality reagents for microscopy, poor workplace environment, and lack of well-trained personnel who are able to accurately prepare and read blood slides [4]. Several interventions such as refresher training in malaria microscopy have been rolled out in order to improve the quality and accuracy of malaria microscopy in Africa [14–16], however, both evaluations and interventions have concentrated on the public sector leaving a gap in the private sector. The private sector plays an important role in increasing access to case management with more than 60% of febrile patients first seeking care in private facilities [17]. In order to achieve the “total health system” approach in addressing challenges in malaria diagnosis, strategies therefore need to be designed to fit both the public and private sectors. This study evaluated the accuracy of blood smear microscopy and the factors associated with inaccurate microscopy in private health facilities of Entebbe Municipality in Uganda.

## Methods

### Study design

This was a cross-sectional study conducted between April and May 2018 in private health facilities that routinely used microscopy for laboratory diagnosis of malaria in Entebbe Municipality.

### Study setting

Entebbe municipality is an urban town located in Central Uganda on a peninsula adjacent to Lake Victoria. It is approximately 37 km, southwest of Kampala, the capital city. The municipality has 36 private health facilities of which 20 have capacity to perform malaria microscopy. Malaria transmission is low in Entebbe, with the prevalence of parasitaemia estimated at 1% by microscopy in children under 5 years according to the 2018–2019 malaria indicator survey [18]. Two peaks (in May and November) of malaria transmission coinciding with peak rainfall patterns are observed in the area [19]. According to the District Health Information System-2 (DHIS2), there are 7 public health facilities (02 at a hospital level, 03 Health Centre III and 02 Health Centre II) in Entebbe Municipality. In 2020, about 71,842 patients were registered at Outpatient Departments (OPD) of these health units. There were 7420 suspected malaria cases of which 1050 (14.2%) tested positive for malaria.

Like the rest of Uganda, the main cause of malaria is *Plasmodium falciparum.* The main malaria control measures in the area include malaria case management with artemisinin-based combined therapy and use of long-lasting insecticidal nets (LLINs). Entebbe has had three rounds of free LLINs distribution (in 2014, 2017, January 2021).

### Study population and procedures

Information on private health facilities in the municipality was collected from the municipal offices and all facilities on the list were visited to establish if they used microscopy for malaria diagnosis. Permission to conduct the study was sought from the health facility heads at facilities with active microscopy during the study period. In all facilities where permission for the study was granted, data was collected over the study period from the laboratory personnel conducting microscopy and from patients fulfilling the inclusion criteria at exit. A health facility assessment was also conducted for all participating facilities.

For exit interviews, participants were included in the study if they; (1) had been referred to the laboratory for microscopy and had their results recorded in their medical form, (2) provided written informed consent to participate in the study (for adults) or had consent provided by their guardian (children under 18 years), (3) provided assent to participate in the study for participants aged 8–17 years. During the exit interview, study personnel stationed daily at the participating clinics collected information about the patient’s age, history of fever, temperature, main complaints and if they used a bed net the night prior to the survey. Facility-based blood smear results were recorded from the participant’s consultation notes, and a second (research) blood slide was obtained by finger-prick by the study personnel for later staining/reading. All research smears were collected prior to initiation of the recommended treatment. All research smears were air dried and fixed at the facility and subsequently transported to the malaria reference laboratory (Makerere University-University of California San Francisco Molecular Laboratory—Mulago) for staining and reading.

### Laboratory procedures

All research smears were stained with 2% Giemsa for 30 min and read by expert microscopists who were blinded to the facility-based results. Thick smears were evaluated for presence of parasites. Parasite densities were determined by counting the number of parasites per 200 leukocytes (or per 500, if the count was less than 10 parasites per 200 leukocytes), assuming a leukocyte count of 8000 cells/µl. Asexual parasitaemia of any level was reported as positive and a smear was considered negative after reviewing 100 high powered fields. All smears were examined by two independent and expert microscopists at the reference laboratory. A third expert microscopist resolved any discrepancies arising from the results of the two microscopists. The expertise level of the microscopists according to the WHO competency assessment protocol is estimated to be level III.

### Data management and analysis

Data were entered and cleaned using Epi Info software version 7.1.5 and exported to STATA version 14 (College Station, TX: Stata Corp LP) for analysis. Measures of diagnostic accuracy (sensitivity, specificity, positive predictive value and negative predictive values) were calculated using expert microscopy as the gold standard. Key study metrics were defined as follows;

1. Sensitivity: The proportion of test results that were positive by both expert and facility-based results divided by the total number of test results that were positive by expert microscopy.
2. Specificity: The proportion of test results that were negative by both expert and facility-based testing, divided by the total number that were negative by expert microscopy.
3. Positive predictive value: The proportion of test results that were positive by both expert microscopy and facility-based testing, divided by the total number of tests that were positive by the facility-based testing.
4. Negative predictive value: The proportion of test results that were negative by both expert microscopy and facility-based testing, divided by the total number of tests that were negative by the facility-based testing.
5. Kappa Statistic was defined as Observed agreement minus Expected agreement divided by 100—expected agreement. This metric was used to estimate the level of agreement between the readers for the facility-based smear and the gold standard microscopists.

Results were categorized as accurate diagnosis (similar result by both expert and health facility technician) or inaccurate diagnosis (the expert result is different from the health facility-based reading). Generalized linear model was used to determine the factors associated with inaccurate BS microscopy and also account for clustering at the level of both health facility and microscopist. All statistical tests were two-sided with adjusted odds ratios that were presented with their 95% confidence interval (CI) and p-values.

## Results

### Study population

There were 20 (55.6%) private health facilities that had microscopy as the routine malaria diagnostic tool, of which 16 (80.0%) agreed to participate and were enrolled in the study. Detailed characteristics of the study health facilities, laboratory personnel, and study participants are presented in Table 1. Of the 16 enrolled facilities, 37.5% (n = 6) provided only outpatient services while the rest provided both in and outpatient services. The majority of the enrolled facilities (62.5%, n = 10) had more than one dedicated laboratory staff who performed the malaria microscopy tests. During the study period, a total of 23 laboratory personnel stained and read blood smears at the 16 participating facilities. A majority of the laboratory personnel (52.2%, n = 12) had a certificate in medical laboratory as their highest level of education.

**Table 1:** Patient, health facility and laboratory personnel characteristics

Characteristic	n (%)
Health facility characteristics (n = 16)	
Number of laboratory personnel per facility	
1	10 (62.5)
2	5 (31.3)
3	1 (6.3)
Type of services	
Out-patient only	6 (37.5)
Out and in-patient	10 (62.6)
Sufficient lighting in the laboratory	12 (75.0)
Sufficient laboratory space	11 (68.8)
Average malaria smears read by technicians per day	
< 5 patients	4 (25.0)
≥ 5 patients	12 (75.0)
Slide re-used	8 (50.0)
Laboratory personnel characteristics (n = 23)	
Sex (female)	4 (18.1)
Age (years)	
< 30	15 (65.2)
≥ 30	8 (34.8)
Working experience (years)	
< 5	11 (47.8)
≥ 5	12 (52.2)
Qualification	
Lab assistant	12 (52.2)
Lab technician	11 (47.8)
Recent refresher training in malaria diagnosis	10 (43.5)
Patient characteristics (n = 321)	
Sex (female)	174 (54.2)
Age (years)	
< 5	50 (15.6)
5–15	42 (13.1)
> 15	229 (71.3)
Reported bed net use the night prior to visit	273 (85.1)
Fever as a presenting complaint	160 (49.8)

At the 16 study facilities, a total of 412 patients were screened to participate in the study, of which 321 (77.9%) were enrolled. Reasons for exclusion included refusal to provide consent (57.1%, n = 52) or lack of a parent/guardian to provide consent for children under 18 years (42.9%, n = 39). The majority (71.3%, n = 229) were more than 15 years of age and their most common presenting complaint was fever (49.8%, n = 160).

### The accuracy of health facility-based microscopy

The overall test positivity rate was 22.7% (73/321) by health facility-based microscopy and 15% (48/321) by expert microscopy (Table 2). A total of 46 smears were positive and 246 were negative by both health facility-based microscopy and expert microscopy, resulting in a percentage agreement of 91.0% (Kappa = 0.71). Using expert microscopy as the gold standard, the overall sensitivity of health facility-based microscopy was 95.8% and specificity was 90.1%. The overall accuracy of the health facility-based microscopy was 91%.

**Table 2:** Slide positivity rate and accuracy of private health facilities-based microscopy compared to expert microscopy

	All participants
Number tested	321
Slide positivity rate (facility, %)	22.7
Slide positivity rate (expert, %)	15.0
Sensitivity	95.8% (85.7–99.5%)
Specificity	90.1% (85.9–93.4%)
Positive predictive value	63.0% (50.9–74.0%)
Negative predictive value	99.2% (97.1–99.9%)

**Fig. 1:**
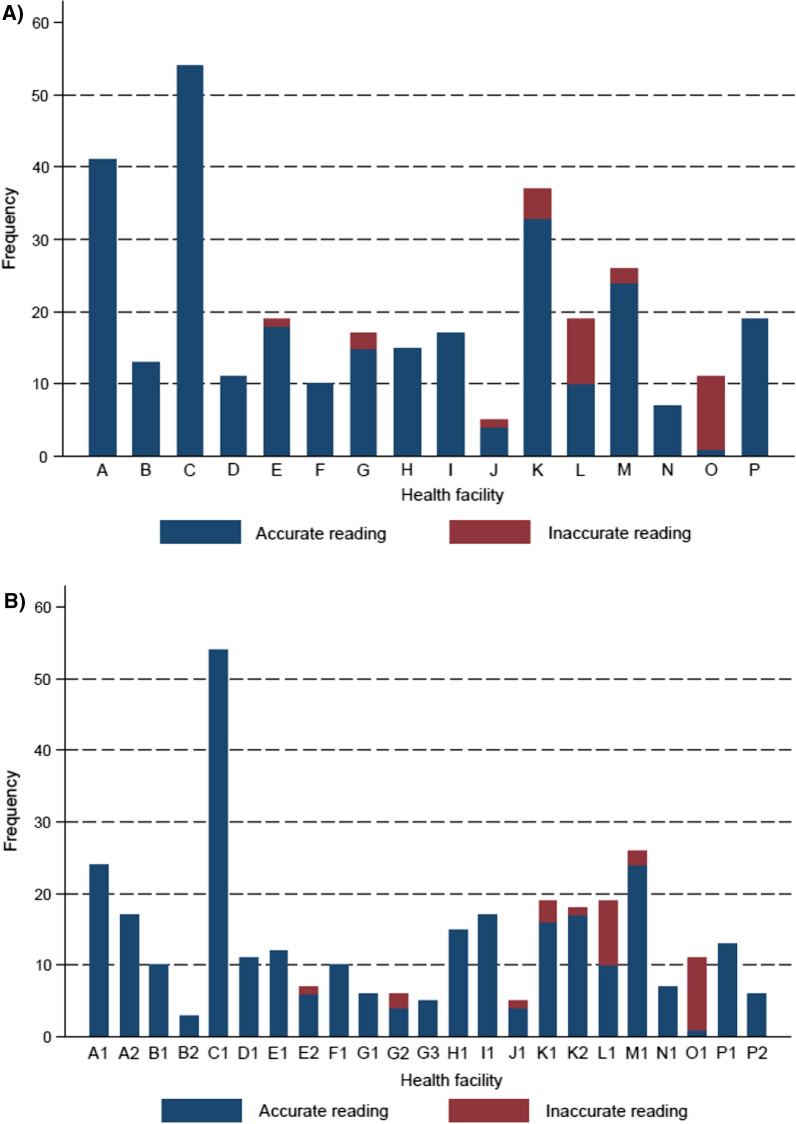
**A** Bar charts showing frequency of accurate facility-based smear readings (blue bars) and inaccurate readings (red bars) at the facility level. **B** Bar charts showing frequency of accurate facility-based smear readings (blue bars) and inaccurate readings (red bars) at the laboratory personnel level

Out of the 321 total smear readings at the facility, 29 readings were inaccurate. The negative predictive value was very high (99.2%), meaning that malaria was missed in only 2 of 248 slides read as negative at the health facility. However, the positive predictive value was relatively poor (63.0%), meaning that 27 of 73 people diagnosed with malaria at the health facility may not actually have malaria. Looking at the risk of inaccurate diagnosis at the level of the health facility and laboratory personnel, the majority of the errors in smear readings were made at just 2 facilities, and each of these facilities had only one laboratory personnel (Fig. 1A, B).

### Factors associated with inaccurate health facility-based malaria microscopy

A total of 29 (9.0%) health facility-based results were inaccurate. False negative and positive results were 2 and 27, respectively. Participants whose smears were examined by a laboratory technician who had worked (read smears) for less than 5 years were more likely to have inaccurate result compared to those examined by a technician with 5 or more years of experience (aOR = 9.74, 95% CI 1.06–89.5, p-value = 0.04). In addition, participants whose smears were read by a technician whose facilities were examining less than 5 smears a day were more likely to have inaccurate smear result than those where the technician were examining 5 or more smears a day (aOR = 38.8, 95% CI 9.65–156, p-value < 0.001). Laboratory personnel who reported not having refresher training in malaria microscopy in the last 5 years had higher odds of having an inaccurate result (aOR = 2.87, 95% CI 0.22–37.5), although the association was not significant (p-value = 0.42). Details of this are summarized in Table 3.

**Table 3:** Factors associated with inaccurate blood smear malaria microscopy

Variable	Inaccurate result n/N (%)	Univariate analysis		Multivariate analysis	
		OR (95% CI)^a^	p-value	OR (95% CI)^a^	p-value
Age of the patient (years)					
< 5	2/50 (4.0)	Reference group		Reference group	
5–15	8/42 (19.1)	1.75 (0.24–12.8)	0.58	1.57 (0.22–11.3)	0.66
≥ 15	19/229 (8.3)	0.83 (0.14–5.11)	0.84	0.58 (0.10–3.40)	0.55
Sex of the patient					
Female	15/174 (8.6)	Reference group		Reference group	
Male	14/147 (9.5)	1.26 (0.45–3.49)	0.66	1.13 (0.41–3.09)	0.82
Reported bed-net use					
Yes	24/273 (8.8)	Reference group		Reference group	
No	5/48 (10.4)	0.95 (0.22–4.14)	0.95	1.06 (0.25–4.51)	0.94
Age of technician (years)					
≥ 30	2/97 (2.1)	Reference group		Reference group	
< 30	27/224 (12.1)	9.88 (0.51–191)	0.13	0.53 (0.06–4.60)	0.57
Qualification of microscopist					
Lab technician	14/183 (7.7	Reference group		Reference group	
Lab assistant	15/138 (10.9)	1.24 (0.09–16.4)	0.87	0.70 (0.15–3.35)	0.66
Experience of technician (years)					
≥ 5	5/183 (2.7)	Reference group		Reference group	
< 5	24/138 (17.4)	5.26 (0.43–63.8)	0.19	9.74 (1.06–89.5)	0.04
Refresher training in malaria microscopy					
Yes	12/152 (7.9)	Reference group		Reference group	
No	17/169 (10.1)	2.84 (0.20–40.3)	0.44	2.87 (0.22–37.5)	0.42
Patient load in the laboratory					
≥ 5 patients	7/250 (2.8)	Reference group		Reference group	
< 5 patients	22/71 (31.0)	38.3 (2.72–539)	0.007	38.8 (9.65–156)	< 0.001
Lighting in the laboratory					
Insufficient	17/276 (6.2)	Reference group		Reference group	
Sufficient	12/45 (26.7)	13.6 (0.35–527)	0.16	2.55 (0.27–24.1)	0.42
Laboratory space					
Insufficient	2/43 (4.7)	Reference group		Reference group	
Sufficient	27/278 (9.7)	1.21 (0.02–78.5)	0.93	1.33 (0.03–57.4)	0.88
Re-used slides					
Sometimes	14/231(6.1)	Reference group		Reference group	
Always	15/90 (16.7)	7.26 (0.51–103)	0.14	1.27 (0.11–14.7)	0.85

^a^Adjusted for repeated observations at the same health facility and by the same laboratory personnel

## Discussion

The accuracy of diagnostic testing is key in informing malaria case management, however, data on the performance of malaria diagnostics in private health facilities in Uganda is still limited. Accuracy of malaria microscopy and factors associated with inaccurate microscopy in 16 private facilities in Entebbe municipality, Uganda were assessed. The findings from this study show that although the accuracy and negative predictive values of the facility-based microscopy in the participating facilities were very high, the positive predictive value was relatively poor (63.0%), with over one third (1/3) of the patients diagnosed with malaria may actually have no disease. The factors associated with a participant having inaccurate malaria smear results included having the smear read by a technician having less than 5 years’ experience in reading malaria smears and having smears read by a technician whose facility was examining less than 5 smears a day.

Accurate diagnosis of malaria is vital for effective management and control of malaria. In Uganda, microscopy remains the gold standard for malaria diagnosis [3]. Malaria microscopy has advantages over rapid diagnostic tests (RDTs) in that it can be used to differentiate malaria species and quantify the parasitaemia and, therefore, is more informative in terms of the most appropriate care to provide a patient [20]. In this study, microscopy was highly accurate, however, one third of the patients diagnosed as having malaria may actually have no the disease. These results provide some assurance that microscopy is still a reliable tool for detecting patients with malaria that present to the private health sector in Uganda. However, the results also raise concerns that a number of patients diagnosed with malaria in this setting, where most patients are first treated in the country [21] may not actually have the disease, resulting in over-diagnosis of malaria. Overdiagnosis of malaria is of concern as it results in anti-malarial drug misuse which may increase the risk of drug resistance, costs to the patient, and missing the true diagnosis for the presenting symptoms.

Although present, the observed rates of malaria over-diagnosis in this study are still much lower than what has been previously recorded in the country. Outpatient malaria over-diagnosis rates are massive in Uganda, reaching as high as 79% in public health facilities [6, 22]. Some improvement in malaria microscopy has been achieved in the last decade, and this has been attributed to in-service training and continuous support supervision [16, 23]. However, in this study, no in-service training activities have been conducted in the private facilities in the study area in the last 10 years. Therefore, the differences in performance observed in this study could not be attributed to in-service training, but may be due to the facilities taking care of fewer manageable numbers of patients compared to the overwhelming patient loads in public facilities, allowing them time to correctly stain and examine the slides.

It is also important to note that only two of the laboratory technicians were responsible for majority of the inaccurate results. Poor microscopy has been associated with multiple factors including; (1) poor training, supervision, and skills maintenance, (2) poor slide preparation techniques, (3) very heavy workloads, (4) poor condition of the microscope, and (5) lack of quality essential laboratory supplies [24–26]. In this study, the experience of the laboratory technologist conducting the smear reading was significantly associated with having inaccurate results. Experience in this study was through either reading many blood smears a day or having more years of reading malaria smears. Experience has previously been highlighted as an important factor in improving the accuracy of malaria microscopy [27] and the WHO recommends reading of at least 10 slides a month in order to maintain the competence of correctly trained microscopists [28].

The team recognizes some limitations in the study including; (1) the study team was stationed at the participating facilities and conducted exit interview with patients seen in the laboratory. This could have modified the behaviour of the laboratory technologists, such that more attention was paid to the smear reading than what was routinely practiced. Indeed studies have reported that medical personnel often modify behaviour when they are aware that they are being observed [29]. This type of bias was minimized by not revealing to the facility personnel that the disease of interest was malaria but were interested in patients sent to the laboratory. In addition, the study staff spent up to 2 months at the facilities and therefore, it is believed that any change in routine practices that may have occurred at the start of the study may have been reverted by the time the study came to an end, which is what is often observed in similar studies [29]. (2) Entebbe municipality is an urban area with unique characteristics that may not be similar to other regions in Uganda, for example all private facilities in the municipality are private for profit (PFP) health units unlike other regions where there is a mix of PFP and private not for profit (PNFP) facilities and thus limiting the generalizability of results to settings similar to the study area; (3) although parasite densities have been reported to affect the accuracy of microscopy [30], this study did not estimate the parasite densities and therefore unable to establish their contribution to the inaccurate results in this study.

In conclusion, the accuracy of malaria microscopy in the private facilities in Entebbe Municipality was high, although one third of patients diagnosed with malaria did not have the disease. Majority of the errors in smear readings were made by two laboratory technicians, and the main factor associated with inaccurate smear results was low experience in malaria microscopy. In-service training may be sufficient to eliminate inaccurate smear results in this setting. The private facilities in this setting would be ideal model facilities to improve the quality of malaria microscopy in Uganda especially in the public sector, by acting as placement centers as well as having technologists at these facilities work as peer mentors for technologists at other facilities.

## Data Availability

The datasets used and/or analysed during the current study are available from the corresponding author on reasonable request.

## Abbreviations

BS: Blood smear
CI: Confidence interval
DHIS2: District Health Information System-2
LLINs: Long-lasting insecticidal nets
MoH: Ministry of Health
NMCP: National Malaria Control Programme
NPV: Negative predictive value
OPD: Outpatient Department
OR: Odds ratio
PCR: Polymerase chain reaction
PFP: Private for profit
PNFP: Private not for profit
PPV: Positive predictive value
RDTs: Rapid diagnostic tests
SOMREC: School of Medicine Research and Ethics Committee
WHO: World Health Organization
